# Intussusception incidence among infants in the UK and Republic of Ireland: A pre-rotavirus vaccine prospective surveillance study

**DOI:** 10.1016/j.vaccine.2013.06.084

**Published:** 2013-08-28

**Authors:** Lamiya Samad, Mario Cortina-Borja, Haitham El Bashir, Alastair G. Sutcliffe, Sean Marven, J. Claire Cameron, Richard Lynn, Brent Taylor

**Affiliations:** a General and Adolescent Paediatric Unit, University College London, Institute of Child Health, England, United Kingdom; b MRC Centre of Epidemiology for Child Health, University College London, Institute of Child Health, England, United Kingdom; c Paediatric Surgical Unit, Sheffield Children's NHS Foundation Trust, England, United Kingdom; d Health Protection Scotland, NHS National Services Scotland, United Kingdom; e British Paediatric Surveillance Unit, Royal College of Paediatrics and Child Health, England, United Kingdom

**Keywords:** BPSU, British Paediatric Surveillance Unit, NHS, National Health Service, ONS, Office for National Statistics, Intussusception, Incidence, Surveillance, BPSU, Vaccine safety

## Abstract

**Introduction:**

Intussusception, an abdominal emergency in young children, has been linked to a previous vaccine used to prevent rotavirus gastroenteritis. Although this vaccine was withdrawn, recent studies have suggested a potential, very small increased risk of intussusception following the administration of newly developed rotavirus vaccines. We aimed to determine the baseline incidence of intussusception among infants in the UK and Republic of Ireland – prior to the imminent introduction of the rotavirus vaccine into the UK schedule this year.

**Methods:**

Prospective, active surveillance via the established British Paediatric Surveillance Unit (BPSU) was carried out from March 2008 to March 2009. Clinicians across 101 National Health Service (and equivalent) hospitals, including 27 paediatric surgical centres, reported cases admitted for intussusception in the UK and Republic of Ireland. The standard Brighton Collaboration case definition was used with only definite cases included for incidence estimation.

**Results:**

The study response rate was 94.5% (379 questionnaires received out of 401 case notifications). A total of 250 definite cases of intussusception were identified. The annual incidence among infants in the UK and Republic of Ireland was 24.8 (95% CI: 21.7–28.2) and 24.2 (95% CI: 15.0–37.0) per 100,000 live births. In the UK, the highest incidence occurred in Northern Ireland (40.6, 95% CI: 21.0–70.8), followed by Scotland (28.7, 95% CI: 17.5–44.3), England (24.2, 95% CI: 20.9–27.9), then Wales (16.9, 95% CI: 6.8–34.8). In England, regional incidence was highest in London and lowest in the West Midlands. By age, the highest incidence (50.3/100,000 live births, 95% CI: 33.4–72.7) occurred in the fifth month of life (for England). A seasonal trend in the presentation of intussusception was observed with the incidence significantly (*p* = 0.001) increased during winter and spring.

**Conclusion:**

The baseline rates obtained in this study will inform rotavirus vaccine-safety policy by enabling comparison with post-introduction incidence.

## Highlights

• The pre-rotavirus vaccine incidence of intussusception among UK and Irish infants was 24.8 and 24.2/100,000 live births.
• The highest incidence (50.3/100,000 live births) occurred in the fifth month of life (for England).
• A seasonal trend in intussusception was observed with the incidence significantly increased during winter and spring.
• Baseline rates will inform rotavirus vaccine-safety policy by enabling comparison with post-introduction incidence.

## 1 Introduction

Intussusception is the most common cause of acute bowel obstruction in children aged less than two years [1,2]. The condition was causally linked to a previous vaccine for preventing rotavirus gastroenteritis in infants [3], followed by withdrawal of this vaccine from the routine immunisation schedule among US infants in 1999 [4]. Subsequently, new rotavirus vaccines have been developed and although initial studies did not show an increased risk of intussusception [5,6], some evidence of a potential, very small elevated risk has recently been found particularly after the first dose of these vaccines (possibly 2/100,000) [7,8]. However, both these studies emphasised the strong evidence of protection offered by these vaccines against rotavirus gastroenteritis – these health benefits as reflected by the number of deaths and hospitalisations prevented from vaccination, substantially outweighed the potential, very small number of intussusception cases that were found to be attributable to vaccination [8].

To continue monitoring the safety of the newly developed rotavirus vaccines, studies worldwide have estimated the baseline incidence of intussusception in order to evaluate more rapidly any adverse event reports following the introduction of these vaccines. Overall, studies in Europe and America have reported the baseline incidence in infants to be ≤60 cases per 100,000 [9–15]. This compares to relatively higher rates (>60 to 302/100,000) reported from Oceania [16,17] including the Far East [18–22], with lower rates (<30/100,000) from south-east and central Asian countries [23,24].

Although genetic, dietary and environmental factors can place some infants at a higher risk for intussusception; a host of other factors including differences in study methodology, availability of diagnostic and specialist services, and access to these services might contribute to the observed variation in the global, baseline incidence.

Although developing countries face the greatest impact of the burden of rotavirus diarrhoea in children under five [25,26], a large number of hospitalisations and clinic visits due to rotavirus infections occur in developed countries including the UK [27]. The UK Department of Health has recently announced the introduction of a new rotavirus vaccine (Rotarix^®^; GSK vaccines) into the UK infant immunisation schedule in 2013. With an 85% efficacy against severe rotavirus gastroenteritis [5], this oral vaccine which will be given to infants in two separate doses with other routine vaccines, is predicted to reduce the burden of severe rotavirus diarrhoea by 70% in England and Wales [28].

The only UK population-based study on intussusception was carried out nearly twenty years ago (1993–1995) and used retrospective, routinely collected data for England [29]. This is the first prospective, nationwide surveillance study to determine the baseline incidence of intussusception in infants in the UK and Republic of Ireland. The study further provides pre-vaccination rates by month of life including the association of seasonal patterns with intussusception.

## 2 Methods

Prospective, active surveillance of intussusception presenting in the first year of life was carried out between 1st March 2008 and 31st March 2009. The established British Paediatric Surveillance Unit (BPSU) reporting system was used in joint collaboration with the British Association of Paediatric Surgeons.

Inclusion criteria were infants admitted with suspected or confirmed intussusception during the study period in National Health Service (NHS) and equivalent hospitals across the UK and Republic of Ireland, aged less than 12 months at the time of admission. This was the first BPSU study to involve paediatric surgeons in addition to the established participation of paediatricians in case reporting.

The BPSU cards were sent monthly to paediatricians and paediatric surgeons requesting them to notify cases of intussusception meeting a standard case definition [30]. Clinicians were then contacted with a brief study questionnaire on the epidemiology and clinical features of intussusception for each notified case.

The study response rate was based on the number of completed questionnaires (including duplicates) returned by clinicians as numerator and the total number of case notifications as denominator. Duplicates were identified using the unique NHS number for England and Wales. The Community Health Index, the Health and Social Care Number and patient/chart numbers along with date of birth and gender were used to identify duplicates for Scotland, Northern Ireland and Republic of Ireland.

Cases were then classified according to internationally agreed and validated Brighton Collaboration Criteria as definite (level 1), probable (level 2) or possible (level 3) [31,32]. Statistical analyses were restricted to definite cases, and also excluded readmission episodes and overseas patients.

The study was approved by the Wandsworth Research Ethics Committee (reference 07/Q0803/62) and the National Information Governance Board (PIAG/BPSU 2-5(FT1)/2007).

### 2.1 Statistical methods

Incidence rates were calculated using definite cases as numerator while the denominator was the total number of live births in England, Wales, Scotland, Northern Ireland and Republic of Ireland [33–36]. Incidence estimates were annualised and expressed per 100,000 live births with 95% confidence intervals. Poisson regression models were fitted to analyse variation in incidence across English regions. GP surgery postcodes were used as a proxy for the child's residential address; these were also used to associate the child to one of the nine English regions or other UK countries.

For England, baseline rates were also obtained by month of life using the number of definite cases (for each month) as numerator. The denominator was obtained from the Office for National Statistics (ONS) by linking births and deaths using the NHS numbers in the cohort of babies born in March 2008 and followed through subsequent months.

Ethnicity data (obtained from clinicians via case-notes) were collected using a standard classification [37]. The annual incidence by ethnic group was calculated for England and Wales using the ONS number of live births for each ethnic group (reported by mother) as denominator.

To study the possible effect of seasonality (of intussusception) on incidence for the UK and Republic of Ireland, the number of definite cases according to each admission month was used as numerator and live births by month of occurrence (UK and Ireland) as denominator. Cosinor models were used to analyse annual seasonal patterns assuming that the monthly frequencies occurred at mid-month [38].

SPSS (version 17, SPSS Inc., Chicago, IL, USA) and the *R* computing environment (version 2. 13.1, R Foundation for Statistical Computing, Vienna, Austria) were used for statistical analyses.

## 3 Results

**Fig. 1:**
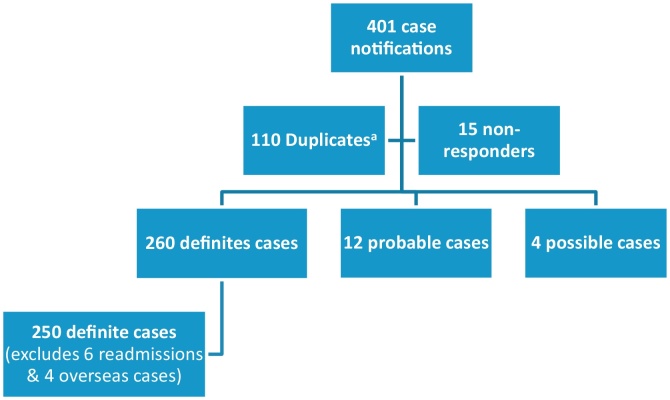
Study profile. ^a^Seven duplicates identified at BPSU card level but questionnaires were not returned for these 7 duplicate cases resulting in 103 (instead of 110) duplicate study questionnaires returned. These patients were initially notified (via BPSU orange cards) by ≥2 paediatricians (from the same hospital) but only one questionnaire was returned for each case.

A total of 101 hospitals from all over the UK and Republic of Ireland reported cases in this study. This included clinicians reporting from 87 paediatric medical and 27 paediatric surgical units (with some duplicate reporting from either different hospitals or from paediatric medical and surgical units of the same hospital). Of 401 cases notified to the BPSU, we received 379 (94.5%) completed questionnaires (including 103 duplicate questionnaires). The remaining 7 out of the 110 identified duplicates occurred at the BPSU card notification level (without any questionnaires returned for these 7 cases). A total of 250 definite cases were thus obtained for analyses after excluding 6 readmissions 4 overseas patients, 12 probable and 4 possible cases (Fig. 1).

Of 250 definite cases, nearing two-thirds (64.8%) of the cases were boys with a male:female ratio of 1.8:1. The median age was 6.2 months (interquartile range: 4.2–8.5 months) with the most frequent occurring age of 5.4 months. Among ethnic groups (243 cases with 7 missing), White British infants comprised the highest percentage (168/243, 69.1%) followed by Other White (28/243, 11.5%) and Black African (11/243, 4.5%) infants.

The annual incidence in the UK was 24.8/100,000 (95% CI: 21.7–28.2) and that of Republic of Ireland was 24.2/100,000 (95% CI: 15.0–37.0). The highest incidence was observed in Northern Ireland followed by Scotland, England and then Wales. In England, London showed the highest incidence while the lowest occurred in the West Midlands region (Table 1). Using Poisson regression and with London as reference category, incidence was found to be significantly lower for the South East (*p* = 0.005), East Midlands (*p* = 0.04) and West Midlands (*p* = 0.01), but not for the remaining English regions (results not shown in Table 1).

Of 190 definite cases in England, the lowest incidence was observed in infants aged less than 2 months, after which the incidence increased reaching a peak in the 5th month followed by an overall decline in the 10th and 11th months of life (Table 2).

Annual baseline rates were obtained by ethnic group for England and Wales (197/250 confirmed cases, 7 missing). The annual incidence among White British infants was 28.1 per 100,000 live births (95% CI: 23.7–33.2). For the remaining ethnic groups, the numbers were low with associated wide confidence intervals (Table 3). Using White British as reference category, Poisson regression analysis was performed to compare baseline rates by ethnic group. The White Other was the only ethnic group, which was significantly (*p* = 0.04) different compared to White British infants (results not shown in Table 3).

**Fig. 2:**
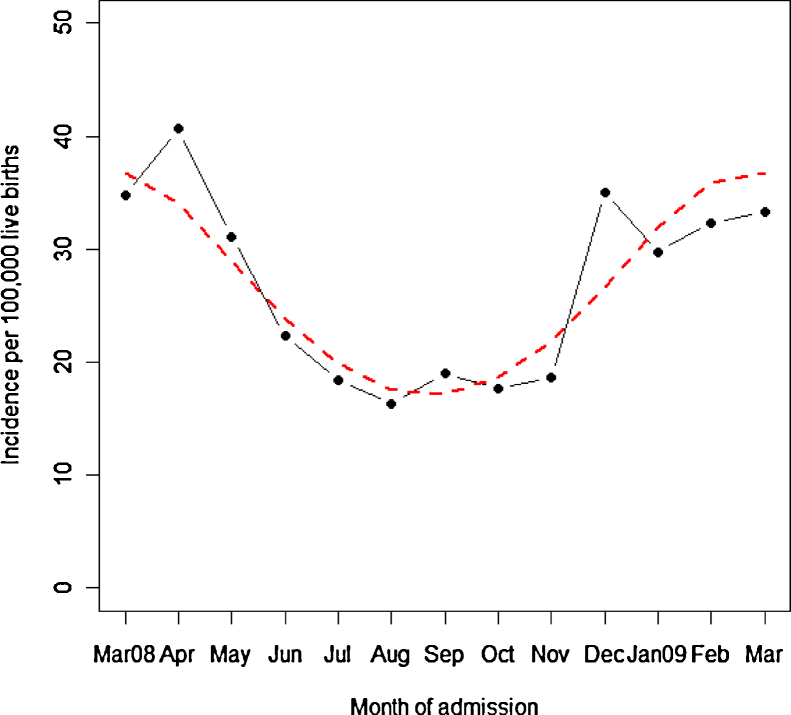
Incidence by month of admission – UK and Republic of Ireland (the dashed line indicates the predicted values from the cosinor model).

A seasonal pattern in the presentation of intussusception in the UK and Republic of Ireland was observed with incidence significantly increasing during winter and spring (*p* = 0.001, Fig. 2).

## 4 Discussion

This is the first study providing current baseline incidence of intussusception prior to the introduction of the rotavirus vaccine into the UK vaccine schedule.

The baseline rates obtained for the UK (24.8/100,000) and Republic of Ireland (24.2/100,000) were lower than those estimated by other European countries with active surveillance systems, such as Switzerland (56/100,000) and Germany (60.4/100,000) [9,10]. Consistent with these National studies, we used the standard Brighton Collaboration case definition for intussusception. We undertook rigorous methods to exclude duplicates. However it is possible that the lower rates observed in our study might reflect some underreporting. There are other methodological differences between the studies; for example, we used definite cases only, whereas in the Swiss study probable cases were also included for incidence estimation [9].

The monitoring of vaccine safety usually involves passive or active surveillance. While underreporting can occur in active surveillance systems which rely a lot on clinical interest and involvement, underestimation is also possible in passive surveillance systems due to coding/misclassification errors or by not including study subjects treated in outpatient/short stay settings [9,11,12]. However, passive systems can also overestimate rates by misclassifying cases, for example, including suspected only cases which have not been clinically confirmed or which do not meet standard case definition criteria [11,17].

There was variation in rates by English region (from 34.2 in London to 15.5 in West Midlands). There might be geographic and/or environmental variation in incidence, but underreporting in a few paediatric surgical centres could also explain the lower rates. A high number of paediatric surgical centres in some regions, for example in London, with patient cross-over to/from nearby regions could also have contributed to the varying rates.

Consistent with previous research, the proportion of boys with intussusception was higher than girls and the median age of 6.2 months fell within the described peak affected ages – the 4th–8th months of life [2,11,17,39,40]. The lowest incidence was observed in the first two months followed by a peak (50.3/100,000 live births) in the fifth month of life (for England). Reasons for this finding may include changes in feeding practices affecting the infant gut [41], maturation of lymphoid tissue or a decline in maternal antibodies against infectious agents possibly associated with intussusception. Along with previous research [11,40,42], these rates provide valuable data to evaluate any age-related change in incidence following the introduction of the rotavirus vaccine among infants in England.

Among ethnic groups in England and Wales, high incidence rates were observed in Black Caribbean and African infants. Although the numbers were small and fell short of statistical significance (except for ‘Other White’) when compared to White British infants, our finding is comparable to US studies in which significantly higher incidence rates were observed in non-Hispanic Black infants compared to non-Hispanic White infants [11,40].

Although most studies have not conclusively identified any distinct seasonality of intussusception [9,10,16,18,40,42], we found a significant seasonal trend with a higher incidence in winter (December–February) and a peak in spring (March–May). In the UK and other European countries, the season of rotavirus gastroenteritis extends from December to April with a peak incidence between January and March [43] including the spring (March–May) quarter [44]. Based on these studies, there appears to be an overlap in our finding of seasonality (of intussusception) and rotavirus gastroenteritis in the UK. This finding however needs to be further explored since no particular association has been observed between the inconsistent seasonal pattern of intussusception and distinct seasonality of rotavirus infections [16,17,45–47].

Our estimated incidence for England was less than half of that (66/100,000 infants) seen in the previous English study [29]. In addition to possible reporting differences such as duplicates, coding errors might explain the higher rate estimated in the previous study, which used routinely collected data (for England). However, the previous study was carried out in 1993–1995, and an actual decline in rates among UK and Irish infants may have occurred since then – a finding that has been seen in other countries [11,17,39].

This study provides National UK and Republic of Ireland pre-vaccination rates of intussusception using prospective, active surveillance with a standard case definition. While underreporting is a potential limitation to active surveillance systems, the participation rate of the BPSU reporting scheme has been shown to be about 94% with at least 89% regional coverage [48]. Although paediatric surgeons were involved for the first time in our study, facilitative methods to achieve maximum case reporting were used such as: hospital visits introducing the study methodology, identification of hospital ‘study-leads’ to review cases and identify those that had been missed, reminder letters sent with study questionnaires including self-addressed envelopes and liaison with hospital staff. Such strategies have been shown to be effective in increasing response rates to postal questionnaires [49].

In order to evaluate the completeness of the established BPSU active surveillance system for intussusception in the UK, we aim to compare our study results with routinely collected hospital data (on intussusception) for England.

In conclusion, with the imminent introduction of the rotavirus vaccine in the UK, these baseline rates of intussusception are now available to inform vaccine-safety policy by enabling comparison with post-introduction incidence.

## Figures and Tables

**Table 1:** Annual incidence (95% CI) expressed per 100,000 live births – by English region/UK country and Republic of Ireland (*n* = 250).

Region/country	Number of definite cases	Number of live births	Annual incidence/100,000 live births (95% CI)
*England*	190	724,260	24.2 (20.9–27.9)
North East	6	32,318	17.1 (6.3–37.3)
North West	29	94,884	28.2 (18.9–40.5)
Yorkshire and Humber	16	71,566	20.6 (11.8–33.5)
East Midlands	11	58,362	17.4 (8.7–31.1)
West Midlands	13	77,366	15.5 (8.3–26.5)
East of England	27	76,996	32.4 (21.3–47.1)
London	51	137,806	34.2 (25.4–44.9)
South East	20	111,933	16.5 (10.1–25.5)
South West	17	63,029	24.9 (14.5–39.9)
*Wales*	7	38,235	16.9 (6.8–34.8)
*Scotland*	20	64,312	28.7 (17.5–44.3)
*Northern Ireland*	12	27,312	40.6 (21.0–70.8)
*UK*	229	854,119	24.8 (21.7–28.2)
*Republic of Ireland*	21	80,147	24.2 (15.0–37.0)

**Table 2:** Incidence by month of life – England (*n* = 190).

Month of life	Number of definite cases	Number of live births	Incidence/100,000 live births (95% CI)
<1	2	55,911^a^	3.6 (0.4–12.9)
1	2	55,733	3.6 (0.4–13.0)
2	15	55,688	26.9 (15.1–44.4)
3	26	55,657	46.7 (30.5–68.5)
4	17	55,647	30.6 (17.8–48.9)
5	28	55,642	50.3 (33.4–72.7)
6	24	55,634	43.1 (27.6–64.2)
7	16	55,627	28.8 (16.4–46.7)
8	21	55,625	37.8 (23.4–57.7)
9	25	55,622	45.0 (29.1–66.4)
10	8	55,619	14.4 (6.2–28.3)
11	6	55,616	10.8 (4.0–23.5)

a Live births in March 2008 (0 month) and remaining cohort (accounting for subsequent cohort deaths), by month of occurrence.

**Table 3:** Incidence by ethnic group – England and Wales (*n* = 197).^a^

Ethnic group	Number of definite cases	Number of live births (by ethnic group)	Annual incidence/100,000 live births (95% CI)
White British	140	459,491	28.1 (23.7–33.2)
White Other	7	51,166	12.6 (5.1–26.0)
Asian – Indian	2	20,258	9.1 (1.1–32.9)
Asian – Pakistani	10	27,637	33.4 (16.0–61.4)
Asian – Bangladeshi	0	9522	0 (0–35.8)
Black Caribbean	5	7491	61.6 (20.0–143.8)
Black African	10	24,052	38.4 (18.4–70.6)
All Others^b^	16	64,003	23.1 (13.2–37.5)

a Missing: 7.

b All Others (for live births by ethnic group – England and Wales) include: Chinese, Other Asian, Other Black, Other, and all Mixed groups.
